# A Signature of Genomic Instability Resulting from Deficient Replication Licensing

**DOI:** 10.1371/journal.pgen.1006547

**Published:** 2017-01-03

**Authors:** Steven C. Pruitt, Maochun Qin, Jianmin Wang, Dimiter Kunnev, Amy Freeland

**Affiliations:** 1 Department of Molecular and Cellular Biology, Roswell Park Cancer Institute, Buffalo, New York, United States of America; 2 Department of Biostatistics and Bioinformatics, Roswell Park Cancer Institute, Buffalo, New York, United States of America; Columbia University, UNITED STATES

## Abstract

Insufficient licensing of DNA replication origins has been shown to result in genome instability, stem cell deficiency, and cancers. However, it is unclear whether the DNA damage resulting from deficient replication licensing occurs generally or if specific sites are preferentially affected. To map locations of ongoing DNA damage in vivo, the DNAs present in red blood cell micronuclei were sequenced. Many micronuclei are the product of DNA breaks that leave acentromeric remnants that failed to segregate during mitosis and should reflect the locations of breaks. To validate the approach we show that micronuclear sequences identify known common fragile sites under conditions that induce breaks at these locations (hydroxyurea). In MCM2 deficient mice a different set of preferred breakage sites is identified that includes the tumor suppressor gene Tcf3, which is known to contribute to T-lymphocytic leukemias that arise in these mice, and the 45S rRNA gene repeats.

## Author Summary

Many RBC micronuclei result from double strand DNA breaks that give rise to acentromeric chromosomal fragments that fail to incorporate into nuclei during mitosis and consequently remain in the cell following enucleation. Here, RBC micronuclear DNA is sequenced (Mic-Seq) to define the locations of breaks genome-wide and this assay is used to study ongoing genome instability resulting from insufficient DNA replication origin licensing. Using a mouse model, we show that there is increased instability at discrete sites across the genome, which include genes that are recurrently deleted in the T-lymphocytic leukemias that eventually arise in these mice. Mic-Seq may provide an effective means of predicting locations that are susceptible to genetic damage and these predictions may have prognostic value.

## Introduction

Licensing of DNA replication origins begins early during the G1-phase of the cell cycle when ORC and CDC6 recruit CDT1-MCM2-7 to the chromatin [reviewed in 1]. MCM2-7 is a heterotypic hexameric complex that is the replicative helicase and, once loaded, its association with the DNA is stable until it becomes active during S-phase. Licensing is restricted to the G1-phase of the cell cycle to prevent endo-reduplication of the DNA by relicensing already duplicated DNA during S-phase. Many more sites are licensed with MCM2-7 complexes than are typically used in S-phase and these dormant origins are thought to serve as a backup system for recovering replication in the event of replication fork stalling or collapse [2–3]. The importance of this system of backup origins for maintaining genome stability is demonstrated by the observation that mice in which MCM2-7 proteins are deficient or compromised develop normally but exhibit increased genome instability, high rates of cancer and stem cell deficiencies [4–6].

The cancers that arise in MCM deficient mice can be highly specific to a particular genetic background. For example, >80% of hypomorphic MCM4 (Mcm4^Chaos3/Chaos3^) females succumb to mammary adenocarcinomas when carried on the C3H genetic background on which it was isolated [4]. In contrast when bred into a C57Bl/6J background females are highly prone to histiocytic sarcoma [7]. Similarly, MCM2 deficient (Mcm2^IRES-CreERT2/ IRES-CreERT2^) mice exhibit near to 100% penetrance of T-lymphocytic leukemia (TLL) within 5 months on the 129Sv background on which they were constructed [5] but a broader spectrum of tumor types with more variable latencies in a mixed 129Sv/BALB/c genetic background [8].

The genetic lesions that occur in tumors arising in MCM deficient or hypomorphic mice are largely copy number alterations (CNAs) with a preponderance of deletions averaging 400–500 kbp in size [9–10]. Several recurrent deletion sites are found in TLLs from MCM2 deficient mice where, on the 129Sv genetic background, the *Tcf3* gene on Chr10 is affected in all tumors examined [9]. *Tcf3* is a transcriptional regulator that plays a central role in T-cell differentiation and prior studies have shown that loss of *Tcf3* is sufficient to drive TLL in mice [11]. Importantly, the *Tcf3* gene lies within a region of Chr10 that shows preferential loss of origin function in MEFs from MCM2 deficient mice [12].

Replication licensing may become limiting due to genetic deletion of *Mcm2-7*, which occurs in many tumors [12], oncogenic stress [1] and during stem cell aging [13]. It is not known if under these conditions genome instability is elevated generally across the genome or whether specific locations are at increased risk; although loss of MCM expression in aging hematopoietic stem cells (HSCs) has been correlated with nucleolar associated DNA damage foci [13]. In the present study we use micronuclear DNA sequences to show that the region of Chr10 carrying the *Tcf3* gene is also the site of elevated ongoing genome instability even prior to tumor initiation in MCM2 deficient mice on the 129Sv genetic background. The increased instability at this site predicts the high rate of genetic lesions affecting the *Tcf3* gene and consequent high rate of TLL in these mice. In addition to loss of *Tcf3*, MCM2 deficient mice exhibit genetic lesions within the 45S ribosomal RNA gene repeats clusters on Chrs 12, 16, 18 and 19 consistent with the observed nucleolar associated DNA damage foci in aging HSCs. The present study demonstrates that the consequences of reduced MCM expression on local genome instability is reflected in micronuclear DNA sequences allowing prediction of specific genetic lesions in the etiology of cancer and during aging.

## Results

### Isolation and sequencing of micronuclear DNA (Mic-Seq)

**Fig 1:**
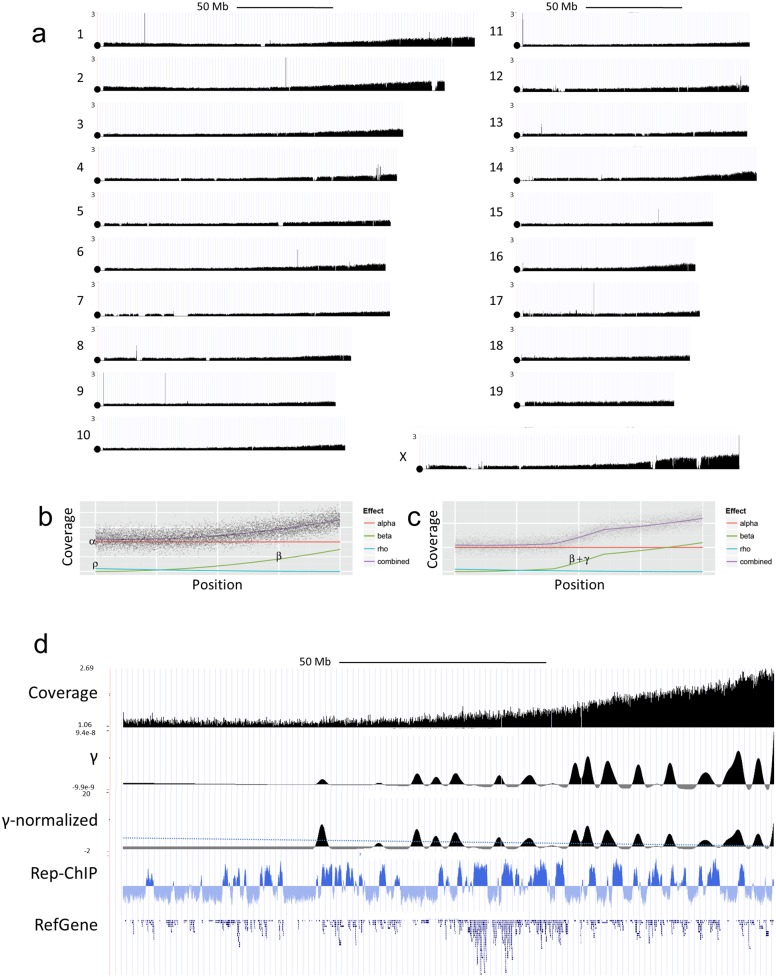
Micronucleus DNA sequences of wild type 129Sv mice. Tagged sequencing libraries were prepared from DNAs isolated from the MN blood fraction of a 6 week old wt 129Sv mouse as described in the text and sequenced using an Illumina HiSeq 2500 sequencer. Panel (a) shows sequence tag distributions for each whole chromosome as indicated in the figure following normalization using the GRN fraction from the same mouse. All mouse chromosomes are telocentric and the centromeric end of each chromosome is marked by a circle. Panels (b) and (c) illustrate parameters defined to describe the observed changes in sequence tag density between and across chromosomes. Panel (d) shows MN sequence tag coverage (coverage), peaks identified on the basis of γ as discussed in the text (γ), the effect of normalizing γ for β and ρ (γ-normalized) where the dotted line represents a cutoff based on 3 s.d. of the estimated variance, and the relationship of these peaks to replication timing (FSU repli-ChIP for MEF [31], FSU ENCODE group) and gene density (RefGene).

To map sites of ongoing genomic instability across the genome we take advantage of the fact that in the hematopoietic system of mammals DNA remnants resulting from genetic damage events during differentiation of hematopoietic stem cells (HSCs) to erythrocytes are retained in the cells as micronuclei following enucleation [14]. To isolate micronuclear DNA, cells from whole blood were first pelleted and washed to remove serum and serum DNAs. Cells were then fractionated into lymphocyte and red blood cell (RBC) plus granulocyte fractions using ficoll-paque. The RBC/granulocyte pellet was then re-suspended, RBCs were lysed using standard RBC lysis protocols and granulocytes were pelleted. DNAs were recovered from three fractions: lymphocyte (WBC), granulocyte (GRN), and the supernatant from the lysed RBCs (containing the RBC micronuclei, MN). Tagged sequencing libraries were prepared from each sample to allow multiplexed high throughput sequencing such that WBC, GRN, and MN from the same animal are run together on the same sequencing lane on an Illumina HiSeq 2500. The resulting sequences were mapped and wiggle files were generated. The sequence coverage, genome wide, from the MN fraction of a wild type (wt) 129Sv mouse is shown in Fig 1a (for comparison, sequence coverage from the WBC and GRN genomic DNAs are shown in S1 Fig panels a and b respectively).

Sequence tag density in the WBC and GRN fractions of wt mice are largely uniform both between and across individual chromosomes. In contrast, in the MN fraction, although the minimal sequence tag density is similar between chromosomes, there is a significant deviation from the average sequence tag density as a function of position across each chromosome. This, in part, reflects the frequency with which different portions of each chromosome are present in micronuclei. Similar patterns are seen in the MN fractions of two different wt mice where the correlation between experimental repeats is 0.988. Several parameters are defined to describe the observed changes quantitatively (Fig 1b and 1c). First, a value α is defined as a base line measure of the representation of each whole chromosome. This parameter is specific to each chromosome and is expected to reflect both the whole chromosome loss rate for that chromosome in MN plus any whole genomic DNA contamination from nucleated cells. The value of α is estimated from the minimum of the sequence tag coverage plot across the chromosome. A value β is defined to describe the observation that sequence tag density increases over all chromosomes as a function of distance from the centromere. This observation is consistent with double strand DNA breaks (DSBs) resulting in exclusion from nuclei of acentromeric DNA fragments from the breakpoint through the distal end of the chromosome [14]. A third phenomenon, which is the converse of β, is referred to as ρ and describes a small increase in sequence tag density as a function of distance relative to the centromere distal telomere of each chromosome and which is observed primarily on the longest chromosomes (Chr1 and Chr2). The mechanism leading to the observed ρ effect is unclear but may reflect mis-segregation or breakage of dicentric chromosomes resulting from errors during DNA repair/translocation [14–15]. The contributions of α, β and ρ to MN sequence tag density, as a function of position on the chromosome, are shown schematically in Fig 1b. In addition to chromosome wide changes, there are discrete locations on most chromosomes that show local increases in sequence tag density that are greater than those that would be expected based on the effects of α, β and ρ. These locations are described by γ which is defined as localized changes in sequence tag density that are cumulative distal to the centromere even through the rate of change returns to levels predicted by β and ρ (Fig 1c). In principle, increased γ values are expected to reflect localized regions (hot spots) of the genome where chromosome breaks occur at increased frequency.

Functions describing β and ρ for wt animals were established using Chr7 and Chr11 since, by inspection, there are few localized increases in sequence tag density on these chromosomes. Both β and ρ are non-linear where the best fit for each is a quadratic function (S5 Fig panel a). It is unlikely that the non-linearity results from a bias in the locations of the initial DSB sites and consistent with this interpretation DSBs that have been repaired by translocation do not exhibit a bias that would lead to β or ρ distributions in micronuclei [15]. One possible explanation is that events occurring during anaphase distort the representation of acentromeric chromosomal fragments in the micronuclear fraction. For example, longer stretches of sister chromatid pairing within the acentromeric region resulting from DSBs more proximal to the centromere may promote non-disjunction and retention of the acentromeric fragment within a nucleus. This could lead to preferential representation of acentromeric chromosome fragments resulting from breaks nearer to the centromere distal ends of the chromosomes consistent with the observed non-linear increase described by β.

To identify localized regions of the genome exhibiting elevated instability, γ values were estimated by determining the rate of the sequence tag coverage change within smoothed and normalized 20 kbp windows genome wide. Although the overall contribution of β and ρ to differential representation of centromere distal and, to a lesser extent proximal ends of whole chromosomes is significant over entire chromosomes, over shorter 20 kb intervals these effects are minimal and the slope of the sequence tag density largely reflects the localized effect γ (e.g. Fig 1d, γ track). Similar to the case for sequence independent breaks, the magnitude of peaks identified by γ is biased towards the centromere distal ends of the chromosomes. This result is expected if the same forces that act to skew the distribution of sequences represented in micronuclei following sequence non-specific breaks also act on chromosome fragments resulting from local hot spots. The effect of normalizing γ peaks using β and ρ values is shown in Fig 1d, γ-normalized track. γ plots show 294 peak locations across the genomes of wt mice. Of these 129 occur in early replicating gene rich regions of the genome and 165 occur in gene poor late replicating, regions of the genome.

### Effect of hydroxyurea on micronuclear DNA sequences

**Fig 2:**
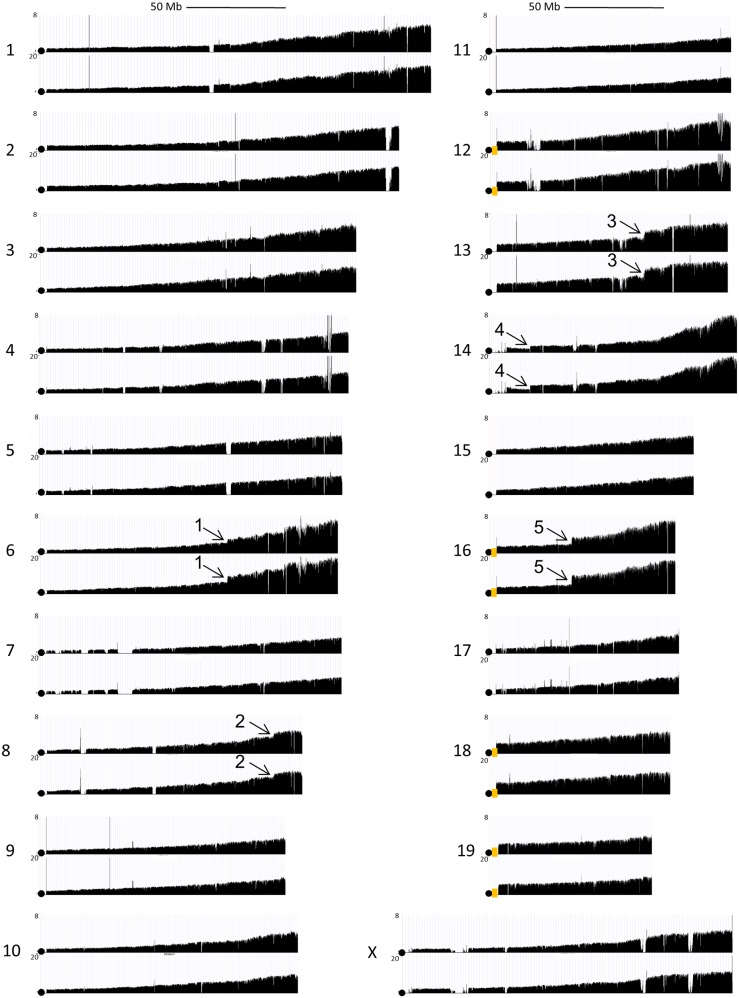
Mic-Seq profiles for HU treated mice. Sequence tag distributions for each whole chromosome as indicated in the figure for two different mice treated with 2 mg/ml HU for 4 weeks. Circles indicate the positions of centromeres and orange boxes indicate the positions of rDNA repeats. Locations (1–5) showing a sharp transition in sequence tag coverage are marked.

**Fig 3:**
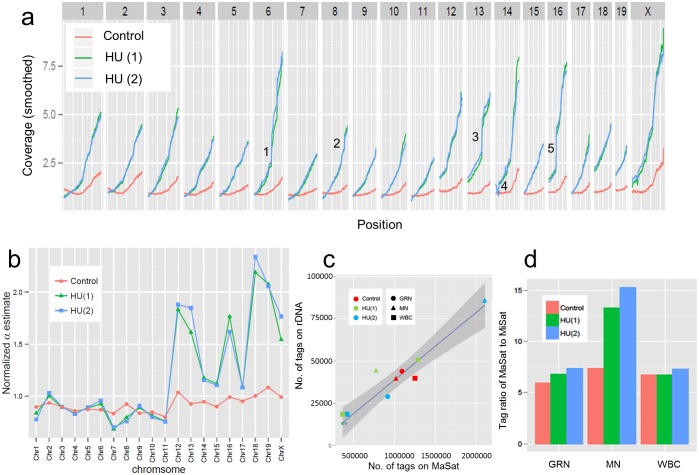
Effect of hydroxyurea (HU) on MN sequence composition. Panel (a) shows MN sequence tag densities across all chromosomes for a wt 129Sv control and 2 repeats of wt 129Sv mice treated with HU (2mg/ml for 4 weeks) where the locations (1–5) marked in Fig 2 are also indicated here. Panel (b) shows α values (y-axis) for each chromosome (indicated on x-axis), normalized to the α-value for Chr3, for the same samples. In panel (c) the number of sequence tags mapping to the 45S rRNA genes are plotted on the y-axis relative to the number of sequence tags mapping to major satellite sequences MaSat on the x-axis for different samples as indicated in the figure. The shaded area indicates the 95% confidence interval. In panel d the ratio of the number of sequence tags mapping the major satellites to the number of sequence tags mapping to the minor satellites is shown for different samples as indicated.

Prior studies have shown that agents that inhibit replication fork progression lead to chromosome breakage at specific locations across the genome referred to as common fragile sites. Characteristics of these sites have been defined where breakage typically occurs at large, late replicating, transcriptionally active genes [16]. To determine if Mic-Seq identifies common fragile sites, Mic-Seq was performed on hydroxyruea (HU) treated mice. HU induces replication stress through inhibition of ribonucleotide reductase which leads to reduced nucleotide pools and increased replication fork stalling [17–18]. To establish an informative dose of HU when administered in the drinking water a titration was performed where mice were assayed at both 1 week and 3 weeks of treatment with different HU doses for micronuclear frequency by FACS (S2 Fig panel a) and at 3 weeks for effects on the levels of various cell types within the blood by CBCs (S2 Fig panel b). The data shows that there is a narrow HU concentration window at which micronuclear frequency is elevated (by ~10 fold) during the interval between 1 and 3 weeks, but which has minimal effects on the frequency of various blood cell types. The short half-life of HU [19] and intermittent dosing resulting from administration in the drinking water makes it likely that only a subset of cells, in various stages of S-phase, is transiently exposed to sufficiently high concentrations of HU to induce damage. The observation that the frequency of micronuclei continues to increase for at least three weeks is consistent with this possibility but also suggests that, once formed, micronuclei are maintained stably in circulating RBCs. Part of the efficacy of Mic-Seq analysis likely depends on the ability to accumulate DNA remnants over a period of time. Mice treated with a dose of 2 mg/ml HU, which resulted in 1–2% micronucleated RBCs, were used for Mic-Seq. Mic-Seq was performed on two mice treated with this dose and an additional untreated wt control. Genome-wide sequence tag distributions are shown in Fig 2 for the MN fraction of both of the HU treated mice and is summarized for all three animals in Fig 3a.

The micronuclear fractions from HU treated mice show a more rapid increase in sequence tag density as a function of distance from the centromere relative to wt mice and the value estimated for β (again using Chr7 and Chr11) is increased ~5 fold (S5 Fig panel a). The increase in β is accompanied by a decrease in the value for ρ suggesting that more frequent chromosomal breaks suppress the mechanism that results in preferential retention of centromere proximal sequences (S5 Fig panel a). HU also affects the distribution of MN sequences across whole chromosomes where Chrs 12, 13, 16, 18, 19 and X show an ~2 fold, increases in α values (Fig 3b). The 45S rRNA gene sequences, which are present on a subset of these chromosomes (see below), also show a modest (~5–20%) overrepresentation in the ratio of rDNA sequence tags to major satellite sequence tags in MN DNA relative to WBC or GRN DNAs from the same mice (Fig 3c). The sequence tag density of major satellite sequences is more strongly enriched (~ two-fold) relative to minor satellite sequences in HU treated mice consistent with preferential induction of breaks in at least a subset of the major satellite sequences by HU (Fig 3d).

**Fig 4:**
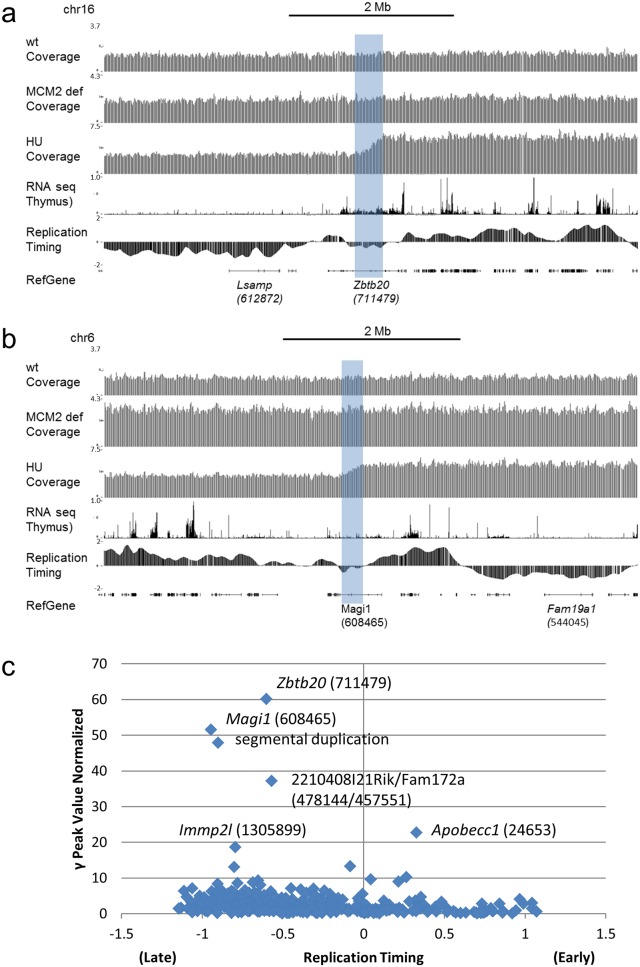
Hydroxyurea sensitive sites are associated with large, late replicating, transcriptionally active genes. Sequence tag coverage for MN from wt, MCM2 deficient, and HU treated mice are shown in panels a and b displayed on the UCSC genome browser for two locations (indicated by the shaded regions) exhibiting large γ values in HU treated mice. Tracks for RefGenes, RNA seq for Thymus ([31], ENCODE LICR group), and replication timing (FSU repli-ChIP for MEL; [31], FSU ENCODE group) are shown for comparison. In each case large genes (size in bp) are marked below the RefGene track. In panel c γ peak height values (normalized for the effects of β and ρ) for MN samples pooled from HU treated mice (y-axis) are plotted against replication timing (FSU repli-ChIP for MEL; [31], FSU ENCODE group) averaged γ peak intervals (x-axis) where late indicates late replicating and early indicates early replicating. Genes (gene size in bp) located at the positions of several of the largest γ peaks are indicated.

Inspection of the micronuclear sequence data from HU treated mice reveals sharp increases in sequence tag coverage distal to discrete locations suggesting that specific sites are disproportionately affected by HU treatment (5 such locations are marked 1–5 in Figs 2 and 3a). Extraction of γ peak values identifies these and additional localized increases in sequence tag densities that include 5 of the 8 molecularly characterized common fragile sites in the mouse (e.g. site 2 is *Wwox*, S2 Fig) and additional large, late replicating, transcriptionally active genes with properties of common fragile sites ([16]; Fig 4a–4c and S2 Fig). In these cases, instability occurs within sub-domains of the genes (Fig 4a and 4b and S2 Fig, panel c) consistent with the fragile site core regions observed in prior studies [15,20].

### Effect of MCM2 deficiency on micronuclear DNA sequences

**Fig 5:**
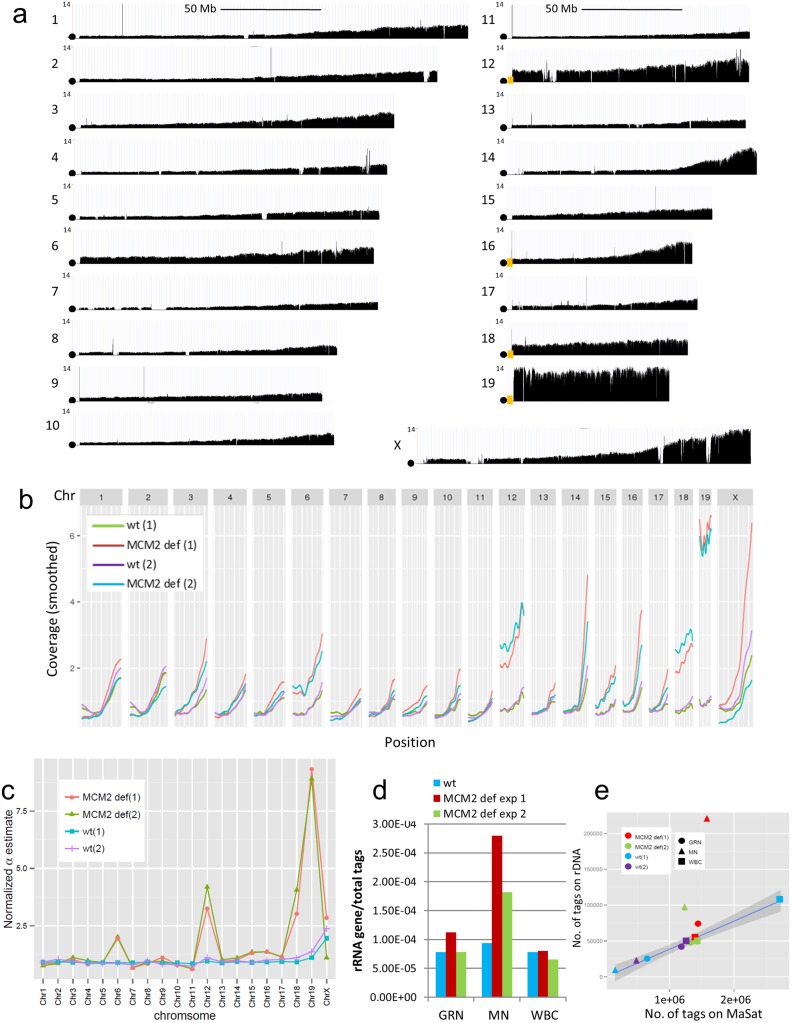
Effect of MCM2 deficiency on MN sequence composition. Panel (a) shows sequence tag distributions for each whole chromosome as indicated in the figure for the MN fraction of an MCM2 deficient mouse. Circles indicate the positions of centromeres and orange boxes indicate the positions of rDNA repeats. Panel (b) summarizes smoothed MN sequence tag densities across all chromosomes for 2 repeats of wt and 2 repeats of MCM2 deficient 129Sv mice. Panel (c) shows α values (y-axis) for each chromosome (indicated on x-axis) for 2 wt and 2 MCM2 deficient mice as indicated in the figure where data sets were normalized to the α-value for Chr5. Panel (d) shows the proportion of sequence tags mapping to the 45S rRNA gene relative to total sequence tags for the granulocyte (GRN), micronuclear (MN) and white blood cell fractions (WBC) fractions from wt (blue) or MCM2 deficient (green or red) mice. In panel (e) the number of sequence tags mapping to the 45S rRNA genes are plotted on the y-axis relative to the number of sequence tags mapping major satellite sequences MaSat on the x-axis for different samples as indicated in the figure. The shaded area indicates the 95% confidence interval excluding the MCM2 deficient MN samples.

To examine the effect of insufficient DNA replication licensing on genome instability, Mic-Seq was performed on mini-chromosome maintenance (MCM) protein 2 deficient mice [5]. MCM2 deficient mice on the 129Sv genetic background exhibit early onset T-lymphocytic leukemia, loss and dysfunction of stem cells, and genome instability evidenced by increased γH2AX in nucleated cells and increased micronuclei in reticulocytes/erythrocytes [5]. The elevated frequency of micronuclei is confirmed in S3 Fig, panels 3a-3c, demonstrating that micronuclei are approximately 10 fold more frequent in RBCs of MCM2 deficient relative to wt animals. For Mic-Seq, MCM2 deficient mice between 5–6 weeks of age, well before the onset of overt disease, were used. MN sequence tag density across the genome of an MCM2 deficient 129Sv mouse is shown in Fig 5a. Two biological repeats of the experiment were performed where the genome-wide correlation between experimental repeats was 0.975. The sequence coverage across all chromosomes is show for both experiments and in comparison to two wt mice in Fig 5 panel b.

Comparison of the sequence tag densities derived from micronuclei of wt and MCM2 deficient 129Sv mice shows that the additional breaks resulting from MCM2 deficiency suppress ρ values and modestly increase β values relative to wt cells (S5 Fig panel a). However, the most pronounced differences are on the α values associated with chromosomes 6, 12, 18 and 19, and to a lesser extent on chromosomes 15 and 16, where these chromosomes are over represented by between ~1.8–12 fold relative to the average sequence tag density across the genome (Fig 5c). The entire mapped region of each of these chromosomes is affected. Unlike the case for HU treatment, the ratio between minor and major satellite sequences is not affected (S3 Fig panel g) suggesting that breaks within the major satellite elements are not responsible.

In C57Bl6 mice, chromosomes 12, 15, 16, 18 and 19 carry nucleoli encoding the 45S ribosomal rRNA gene repeats at centromere proximal positions (NCBI http://www.ncbi.nlm.nih.gov/gene/19791). These are typically composed of 30–40 repeats of an approximately 45 kb repeating unit at each location (additional low copy number rDNA repeats have also been mapped to Chr1, Chr6, and Chr9 in some strains; [21]). One potential explanation for the over representation of the subset of chromosomes seen in micronuclei from MCM2 deficient mice is that rDNA repeats are hypersensitive to reduced replication origin licensing resulting in high rates of double strand breaks within these repeats. Since rDNA repeats are adjacent to the centromeres in these chromosomes, a DSB within an rDNA repeat would render nearly all of the affected chromosome acentromeric.

To determine the locations of rDNA clusters in 129Sv mice, metaphase chromosomes from wt 129Sv MEFs were assayed by fluorescence *in situ* hybridization (FISH) plus spectral karyotyping (SKY) to localize a probe for the 45S ribosomal gene (containing portions of the 18S, 5.8S and 28S ribosomal genes) to specific chromosomes. Results from these studies (S3 Fig panels 3d-3e) localize ribosomal gene repeats to Chrs 12, 16, 18 and 19 in most metaphase spreads from 129Sv mice. In addition, a small subset (2%) exhibit signal consistent with recombination events leading to the presence of rDNA repeats on Chr6 (and in MCM2 deficient MEFS, Chr15). Although these results are consistent with a role for rDNA repeats in the increased representation of specific chromosomes within micronuclei, the relative FISH signal intensity between different chromosomes (19 = 12>18 = 16, S3 Fig panel f) does not correlate with the representation of these chromosomes in MN of MCM2 deficient mice (19>>12 = 18>>16, Fig 5c). However, if the increased representation of the mapped regions of these chromosomes results from DSBs in the rDNA repeats, representation of the acentric regions is expected to be affected by β. Following normalization for β, the representation of different chromosomes in MN is similar to that expected based on rDNA copy number as estimated from FISH (i.e. 19 = 12>18 = 16).

rDNA sequences are over represented by a factor of 2–3 in the MN fraction, relative to the GRN or WBC fractions, of MCM2 deficient but not wt mice (Fig 5d). Further, rDNA sequences are enriched by a factor of 2–5 fold relative to peri-centromeric (major satellite, Fig 5e) and centromeric (minor satellite) repeat sequences in MN of MCM2 deficient mice in comparison with the WBC or GRN fractions of the same mice or all fractions from wt animals. These results support a large increase in the rate of DSBs in at least a subset of rDNA repeats of MCM2 deficient relative to wt mice. Consistent with this interpretation, many of the additional γH2AX foci observed in MCM2 deficient, relative to wt, MEFs are located over nucleoli (S4 Fig panels a-g). Further, short nascent stand analysis shows that a subset of DNA replication origins within the 45S rRNA gene repeats are preferentially affected by MCM2 deficiency (S4 Fig panels h-j).

**Fig 6:**
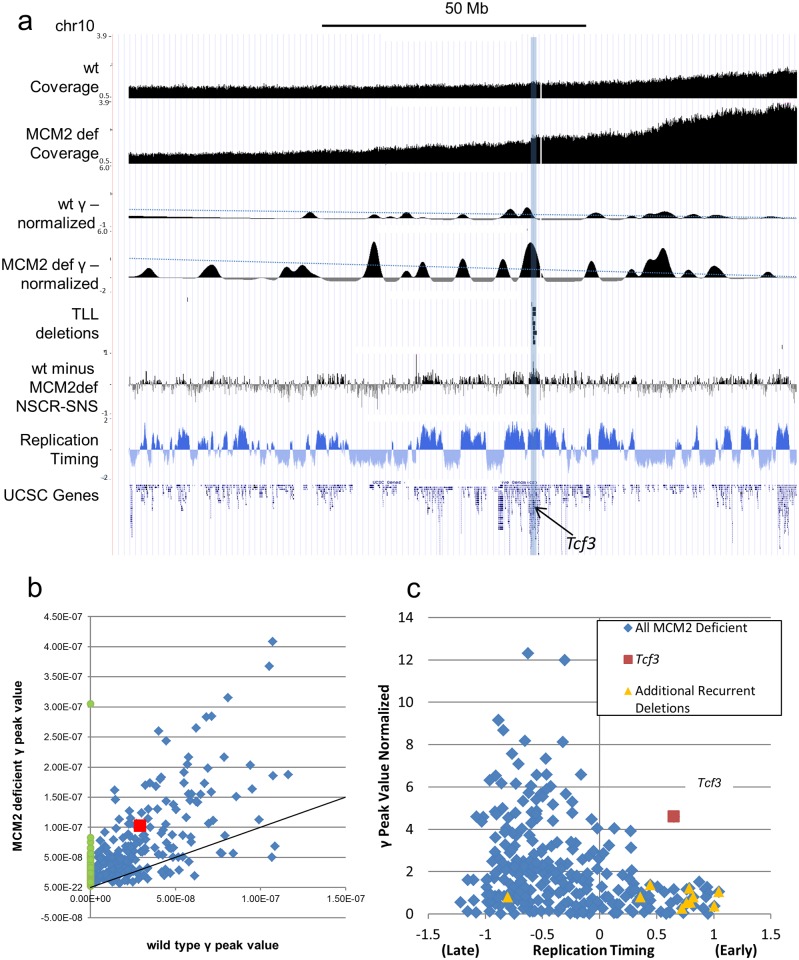
Genome instability hot spots in MCM2 deficient mice. In panel (a) pooled sequence tag data from 3 wt and 2 MCM2 deficient mice are shown where the normalized sequence tag coverage of wt and MCM2 deficient mice and the γ peaks (normalized for β and ρ) derived from these plots (where in each case the dotted line represents a cutoff based on 3 s.d. of the estimated variance) are compared to locations where CNVs occur inTLLs from MCM2 deficient mice [9], a difference plot of short nascent strands from wt minus MCM2 deficient MEFs [12], replication timing (FSU repli-ChIP for MEF; [31], FSU ENCODE group) and gene density (UCSC Genes) across Chr10 as indicated. The shaded region marks the location of recurrent deletions including the *Tcf3* gene. Panel (b) is a scatter plot showing the relative γ peak height values of peaks that are common between MCM2 deficient (y-axis) and wt (x-axis) MN samples (blue diamonds) and peaks that are unique to MCM2 deficient MN samples (green circles). The red square marks the common peak at the location of the *Tcf3* gene and the line shows a 1:1 ratio between MCM2 deficient and wt scales. Panel (c) shows γ peak height values (normalized for β and ρ) for MCM2 deficient MN (y-axis) plotted against replication timing averaged over the γ peak intervals (FSU repli-ChIP for MEL; [31], FSU ENCODE group) where late indicates late replicating and early indicates early replicating. The peak found at the position of the *Tcf3* gene is marked by a red square and peaks at the locations of additional recurrent deletions that are found in TLLs but less frequently than those at *Tcf3* are marked by orange triangles.

To examine the effect of MCM2 deficiency on localized increases in genetic damage in regions other than the 45S rRNA gene repeats, γ values were compared between wt and MCM2 deficient mice genome-wide (e.g. Chr10, Fig 6a). Most locations where peaks are present in the γ plot of MN from wt mice are also represented in MN from MCM2 deficient mice (214 common peaks across the autosomes) but at increased values where the average increase was 2.4 fold (Fig 6b, blue diamonds). An additional 63 peaks are present only in MN from MCM2 deficient mice (Fig 6b, green circles). Prior studies [9] have identified locations of recurrent deletions in T-lymphocytic leukemias (TLLs) arising in MCM2 deficient mice. One location that undergoes deletion in all TLLs of MCM2 deficient mice on the 129Sv genetic background is the *Tcf3* gene on Chr10 and this location is highlighted in Fig 6a. *Tcf3* is required for T-cell differentiation and loss of *Tcf3* results in TLL [11]. This site is also a location at which MCM2 deficiency has a disproportionately strong effect in reducing origin usage as measured by short nascent strand analysis [12]. Increased genome instability is detected by Mic Seq at this site and, of the early replicating regions of the genome, the region containing the *Tcf3* gene shows the highest level of instability genome wide (Fig 6c). It is likely that instability at this site drives a high rate of loss of *Tcf3* resulting in the near 100% penetrance of early onset TLLs in these mice. Smaller local increases in γ values are also found at many of the additional recurrent deletion sites found in these tumors (Fig 6c). However, locations where MCM2 deficiency has the largest effects on representation of sequences in MN, and results in the greatest increases in γ peak values, occur in preferentially in late replicating gene poor regions of the genome that are not the sites of recurrent deletions in TLLs.

## Discussion

Chromosomal fragile sites are genomic locations that are hotspots for genome instability leading to translocations, amplifications and deletions. Such locations were first defined cytogenetically following treatment of cells in culture with agents that impede DNA polymerase (including aphidicolin, hydroxyurea, 5-azacytidine and bromodeoxyuridine) and mapping breaks in banded metaphase chromosomes. Numerous studies have characterized sites that are frequently affected in human and mouse cells and led to identification of a set of locations termed common fragile sites that are affected under conditions of chemically induced replication stress in a high proportion of individuals. These sites have been extensively characterized and are significantly associated with the presence of large, transcriptionally active, and late replicating genes over 300 kbp in size [16]. The frequency of chromosome breaks at these locations is dependent on cell type and the specific agent used to induce replication stress.

Here we have used the representation of different genomic regions in micronuclear DNA sequences to infer the frequency of chromosome breaks in erythroid cells *in vivo*. Many micronuclear sequences result from the presence of double strand DNA breaks that lead to failure of acentromeric portions of chromosomes to segregate to the nucleus during mitosis. Further we take advantage of the fact that micronuclei are retained in maturing RBCs following enucleation in mammals. In contrast to defining fragile sites cytogenetically, harvesting micronuclei from RBCs allows recovery of tens of millions chromosomal remnants each of which defines a breakpoint that can be queried at nucleotide level resolution by high throughput sequencing. Although there is a concern that erythroblasts (just before enucleation) may not reflect the DNA repair and checkpoint responses typical of other cells, application of the Mic-Seq method to define fragile sites in mice treated with hydryoxyurea shows that the method identifies 5 of the 8 molecularly characterized fragile sites previously defined in mouse lymphocytes [22] including those occurring at the *Wwox* and *Immp2* genes. Further sites detected in this study support that the majority of the most sensitive HU induced fragile sites occur within subdomains of the transcribed regions of large (>300 kbp), transcriptionally active, genes similar to prior studies and consistent with the possibility of interference between the transcription and replication machinery under conditions of DNA polymerase inhibition [15–16, 20].

Similar to HU treatment, reduced replication licensing results in genome instability, increased DSBs, and chromosomal deletions and rearrangements [2–5, 9]. The mechanism resulting in this damage is likely to differ from that mediating HU induced damage; however, it has not been previously determined whether similar or different locations across the genome are preferentially affected. In this study we demonstrate that MCM2 deficient mice exhibit a distinct sequence tag distribution profile in Mic-Seq relative to HU treated mice. HU induced fragile sites are not preferentially sensitive to MCM2 deficiency (e.g. Fig 4, panels a and b) and there is little overlap with early replicating fragile sites induced by higher concentrations of HU [23]. Unlike HU treated mice, the locations at which MCM2 deficient mice exhibit localized damage by Mic-Seq analysis are largely locations that are already sensitive in wt cells but become more prone to breakage in MCM2 deficient mice. Differences in the genome instability profiles are likely to reflect differences in the mechanisms by which HU and MCM2 deficiency affect genome stability. Prior studies have shown that, unlike HU or aphidicolin, reducing MCM levels does not affect the rate of DNA polymerization since similar tract lengths of CldU or IdU incorporation are found between wt and MCM deficient cells by DNA fiber analysis [2–3, 8]. However, DNA fiber analysis also shows that MCM deficient cells are unable to initiate DNA synthesis from dormant origins under conditions (HU treatment) that lead to replication fork stalling [2–3, 8] consistent with a reduction in the frequency of licensed origins.

Short nascent strand analysis has shown that origin usage does not decline uniformly across the genome, but rather specific locations lose function preferentially, in MCM2 deficient MEFs [12]. These locations tend to occur in gene rich, early replicating, regions of the genome although they are not exclusive to transcribed regions of active genes and include origins within inactive genes and intergenic regions [12]. In contrast, Mic-Seq localizes sites that are preferentially prone to breakage to subsets of both early and late replicating regions. This result suggests that factors in addition to the degree of reduction in replication licensing affect the rate of chromosome breaks under conditions of MCM2-7 deficiency. However, even in late replicating regions of the genome these factors are not the same as those determining fragile site locations following HU treatment since the locations that become sensitized to breakage by MCM2 deficiency are not known common fragile sites, or large genes generally, and many contain few or no transcribed regions.

Even within early replicating regions of the genome chromosomal breaks detected by Mic-Seq show only a modest correlation with locations where SNS analysis demonstrates that origin usage is most affected. Nonetheless, it is important that many of the locations showing recurrent deletions in the TLLs that arise in MCM2 deficient mice [9] are sites at which there is both a preferential loss of origin function [12] and an increase in DSBs detected by Mic-Seq. In particular, loss of *Tcf3* is sufficient to drive TLL formation [11] and the region carrying this gene shows a strong differential signal between wt and MCM2 deficient mice in both SNS and Mic-Seq analyses. These differences are apparent in samples taken well before tumorigenesis is observed, likely before tumors are initiated, and support that an increased DSB rate detected by Mic-Seq can in some instances predict a high probability of tumor occurrence.

The strongest signals observed by Mic-Seq in MCM2 deficient mice are associated with chromosomes carrying nucleoli and implicate a high rate of DSBs within rDNA clusters as the location of much of the DNA damage, and the source of a large proportion of the additional micronuclei, found in MCM2 deficient mice. This observation confirms and extends prior studies showing that, as mice age, MCM levels are suppressed in HSCs and, coincident with the loss of MCM expression, nucleolar associated γ-H2AX foci accumulate [13]. The presence of unidirectional replication fork barriers [reviewed in 24] may sensitize rRNA gene repeats to loss of licensed replication origins.

These results demonstrate that experimental reduction of MCM proteins in young asymptomatic mice is sufficient to cause a profile of genome instability that predicts at least a subset of chromosomal locations where genetic damage is found in both cancers and during aging. The observation that, unlike HU, many locations where MCM2 deficiency causes instability can already be detected in young wt mice suggests that even under normal conditions the distribution of licensed DNA replication origins contributes to base line levels of chromosomal instability. Although replication stress is widely recognized as a potent cause of genomic instability [1, 25], the present study emphasizes that different mechanisms leading to replication stress have very different consequences for instability at various locations across the genome and can result in very different phenotypic outcomes.

## Methods

### Ethics statement

Animal husbandry programs and protocol reviews are in compliance with NIH, USDA, and New York State Standards. Mice were maintained in facilities covered under NIH assurance #A-3143-01, certified by New York State for the use of living animals, and the USDA APIHS registration as research facility #21–124. The studies were approved by the Roswell Park Cancer Institute Animal Care and Use Committee under Protocols 817M and 876M.

### Mice and treatments

Five to six week old wild type 129Sv and Mcm2 ^IRES-CreERT2/IRES-CreERT2^ (MCM2 deficient) mice were used in studies addressing the effects of MCM2 deficiency. For studies addressing the effects of hydroxyurea (HU), 3 month old wild type 129Sv mice were administered HU continuously in the drinking water at the concentrations indicated in the text. Blood samples were taken by retro-orbital bleed or cardiac puncture.

### Micronucleus assay

For flow cytometric analysis of micronuclei [26] blood samples were fixed in methanol on the day of sample collection and processed for flow cytometry using the Litron MicroFlow plus kit for mouse blood as per the manufacturer’s instructions (Cat. No. 552730, BD Biosciences).

### Fluorescence in situ hybridization (FISH)/spectral karyotyping (SKY)

Combined SKY/FISH was performed on wt or MCM2 deficient mouse embryonic fibroblast (passage 3) by the Roswell Park Cancer Institute SKY/FISH core facility. The rDNA probe for FISH analysis was prepared using Nick Translation Reagent Kit 07J00-001 (Abbott Molecular Inc.) Green-dUTP 02N32-050 (Abbott Molecular Inc.) to fluorescently label a 7109 bp EcoRI fragment from human genomic ribosomal gene DNA containing a portion of the 18S ribosomal RNA gene, the intergenic spacer, the 5.8S ribosomal RNA gene and a portion of the 28S ribosomal RNA gene.

### Blood fractionation and DNA isolation

Between 400–500 μl of whole blood was washed with 10 ml of phosphate buffered saline (PBS) 3 times and re-suspended in 3 ml PBS. The sample was then layered over 2 ml of Lymphocyte Separation Medium (density = 1.077–1.080 g/ml; Mediatech Inc.) in a 15 ml centrifuge tube and spun for 15 min at 800 RPM. Lymphocytes (WBCs) at the PBS-media interphases were collected and placed in a 15 ml tube and washed 3 times with 10 ml PBS prior to pelleting for DNA isolation. Separation media was removed from the red blood cell (RBC)/granulocyte (GRN) pellet in the original tube and cells were washed 3 times with 10 ml PBS and pelleted. The cell pellet was then resuspended in 4 ml of RLF lyse buffer and incubated at room temperature for 5 minutes prior to spinning at 800 RPM for 5 minutes. The supernatant was collected as the RBC micronuclear fraction (MN). The pellet is the granulocyte fraction and was washed with 10 ml PBS and pelleted for DNA isolation. WBC and GRN pellets were re-suspended in 2 ml 1X lysis buffer with 1 ug/ml proteinase K and the RBC fraction was brought to 1X lysis buffer and 1 μg/ml proteinase K using 4X lysis buffer. Samples were incubated at 37°C overnight and extracted with an equal volume of phenol-chloroform-isoamyl alcohol. Nucleic acid was precipitated with isopropanol, washed with 70% ethanol and re-suspended in 100 ul TE buffer. Samples were treated with 2000 units ribonuclease T1 (Invitrogen) and 3 ug ribonuclease A (Invitrogen) for 30 minutes at 37°C followed by phenol-chloroform-isoamyl alcohol extraction and ethanol precipitation of the DNA.

### DNA sequencing and bioinformatics

#### Sequencing

Libraries were prepared for DNA sequencing using Wafergen prep X kits and TruSeq Index tags (Granulocyte, TruSeq Index 4, TGACCA; MN/Reticulocyte, TruSeq Index, CAGATC and white blood cell, TruSeq Index 25, ACTGAT). Paired end 100 nt sequences were generated using an Illumina HiSeq2500. A summary of numbers of mapped sequence tags generated for each sequencing library is given in S1 Table.

*Sequence mapping and coverage generation*: Sequences were aligned to the reference mouse genome (mm9) using BWA [27] with default parameters. Wiggle/bedgraph files were generated from fragments bridged by pair-end sequences using proper paired reads with mapping quality greater than 20 and PCR duplicates removed. Histograms of fragment insert size distribution were generated for all samples, to determine the maximum insert size for coverage file generation.

#### Reproducibility data pooling and windowing

The Pearson correlation coefficient is 0.975 between MCM2 deficient samples 1 and 2, 0.938 between HU treated samples 1 and 2, and 0.969, 0.978, and 0.988 between wt samples 1 and 2, 2 and 3 and 1 and 3 respectively. (ChrX and chrY are excluded in the calculation as not all animals are the same gender.) Based on the extremely high linear correlations, data was pooled for samples from the same experimental condition to reach better accuracy and allow the use of smaller window sizes in subsequent analyses. A window based approach is employed to infer the parameters in the model by counting read pairs in overlapping windows. Proper paired reads with mapping scores greater than 20 are assigned to a window if the middle point of the left-end read is within the window. A 40 kb window was applied where adjacent windows are overlapped by 20 kb and for pooled data, a 20 kb window with a 10 kb overlap is used.

#### Data normalization

Window count data are first normalized against the GRN fraction where deviation in sequence coverage is expected to result from GC bias, other sequencing biases and strain specific bias as the reference genome is not 129Sv. The GRN fraction from the HU untreated control was used as it shows similar bias with MN fractions and windows with read counts outside of three standard deviations are excluded. The data are further normalized to remove any effect related to sequencing depth using scaling factors determined by α estimates. The number of read pairs for each sample is related to parameter estimates as more reads often have better coverage/higher α. α values are estimated using the minimum median value of 1000 consecutive data points, this estimate ignores β and ρ effects and is an over-estimate. Then the median ratio is selected as a base line for data scaling after excluding chromosomes with strong α changes, which include chr 6, 12, 15, 16, 18, 19, and X. The normalization will make any biological effects comparable as they are scaled to the same level. For the pooled data, the following factors are applied: MCM2 deficient (1.742), HU treated (1.000) and wt (2.037).

*γ plots*: To extract γ values, pooled coverage data was windowed and smoothed monotonically. Monotonic smoothing is used to suppress the effect of local changes in sequence tag coverage due to GC bias or other effects that are unrelated to the cumulative changes in coverage expected for γ. For chromosomes exhibiting significant levels of ρ (Chr1 and Chr2 in wt samples), a monotonically decreasing smoothing line is fitted before the minimum median value point while after that point an increasing smoothing line is fitted. R package scam [28] is used to fit the monotonic lines. For chromosomes without a significant ρ effect an increasing smooth line is fitted. Data were normalized to equivalent average tag densities between 20 kb windows and γ for each window is calculated as the slope of the monotonically smoothed and normalized sequence tag density over the window.

γ plots were further normalized by removing β and ρ effects estimated using chr7 and 11. To estimate β and ρ, a quadratic function with 2 quadratic terms for beta and rho separately is fitted using non-linear weighted least-squares method provided by nls R function. Chr7 and chr11 are used for the inference as, by inspection, they have smallest γ effects. To further estimate the accuracy of the estimation, bootstraps of 50% data points without replacement with 1000 repeats were carried out to estimate standard deviation and 95% confidence interval. The effect of α, β and ρ on sequence tag coverage at various positions across a chromosome is then described as α + βx^2^ + ρ(Chr length—x)^2^ where deviation from this relationship is defined as γ. Finally, the distance effects of γ values related to telomeres were normalized by multiplying the corresponding distance and the γ peaks do not show any relationship with genomic locations after normalization.

To establish cutoff values for normalized γ peaks, the γ values (with β and ρ effect removed) on chr11 and chr7 are used to estimate the null distribution and the histograms are shown in S6 Fig. The standard deviations for each treatment group are derived after removing extremely large values (greater than 1e-7). The values calculated from chr11, which are greater and give more conservative estimation, are used. The normalized γ values are obtained by multiplying the distance to telomere with β and ρ effect removed γ values, the cutoff values for significance at each position are similar determined by multiplying the distance to telomere with three times standard deviation for each treatment group respectively. A normalized γ peak value is considered as significant if it’s greater than corresponding cutoff value. It is assumed that chr11 and chr7 have no significant γ values which may not hold as signified by positive tails in the histograms, so the resulting significance is a conservative estimate. A table of normalized start and end values for 3 s.d. cutoffs for all chromosomes is given for samples from wt, HU treated and MCM2 deficient mice in S3 Table where the function is linear on normalized γ-plots.

#### rDNA repeat and satellite DNA sequence coverage estimates

Major satellite (MaSat) and minor satellite (MiSat) sequences [29] were concatenated into a single sequence and along with the 45S rRNA gene sequence [30] appended into the mm9 reference sequence to build the new reference sequence for mapping.

## Data Access

Sequence data from this study have been submitted to the NCBI short reads archive (SRA), (https://www.ncbi.nlm.nih.gov/sra/?term=SRP091564) under accession number SRP091564.

## Supporting Information

S1 Fig WBC and GRN DNA sequences from a wild type 129Sv mouse. Tagged sequencing libraries were prepared from DNAs isolated from the WBC (panel a) and GRN (panel b) blood fractions of the 6 week old wt 129Sv mouse shown in Fig 1 for the MN fraction. Each whole chromosome is shown as indicated in the figures. The MN fraction shown in Fig 1a and the WBC and GRN fractions, shown in panels a and b here, were sequenced on the same lane using an Illumina HiSeq 2500 sequencer.
(TIF)

S2 Fig Effect of hydroxyurea (HU) on MN frequency and blood composition. Wild type 129Sv mice were treated with varying concentrations of HU (n = 3 for each condition) as indicated and assayed for RBC MN frequency at 1 week or 3 weeks of treatment in panel (a) and by CBCs at three week of treatment in panel (b). Panel (c) is a list of genes over 300 kbp in length that contain the largest γ peak regions in HU treated mice. These include sites 1–3 and 5 (indicated in parentheses following the gene name) as marked in Fig 2 (site 4 lies within a segmental duplication on chr14 and is omitted since no gene is present). As shown in the table, the regions of instability identified by Mic-Seq lie within subdomains of the gene bodies. For each gene the gene coordinates, size in bp, replication timing value (FSU repli-ChIP for MEF; [31], FSU ENCODE group), relative transcription (fragments per kilobase of exon per million fragments mapped, FPKM, for two RNA seq replicates of thymus, [31], ENCODE LICR group), the local maximum for the γ value, the γ value normalized for β and ρ, the 3 s.d. cutoff value for the normalized γ value at the gene position, and the coordinates over which the change in slope occurs are given.
(TIF)

S3 Fig Effect of MCM2 deficiency on MN frequency and sequence composition. Panels A and B show 20X images of blood smears from 6 week old129Sv wt (panel a) and MCM2 deficient (panel b) mice stained with acridine orange (green). Arrows indicate micronuclei. Panel c shows RBC micronuclear frequency determined by flow cytometry for wt (blue, N = 2)) and MCM2 deficient (red, N = 2) 6 week old129Sv mice (error bars indicate s.d.). Panel d shows SKY/FISH analysis to identify chromosomes hybridizing to a 45S rRNA gene probe sequence where signal is seen as green (a spectral karyotype pseudo-colored of the larger image is shown in the inset). Chromosomes 6, 12, 15, 16, 18, and 19 are marked by arrows as indicated. Panel (e) shows the number of chromatids exhibiting 45S rRNA gene signal and panel (f) shows the average signal strength per chromatid (for those exhibiting signal) in 13 metaphase spreads (52 chromatids) for each chromosome (error bars indicate s.d.). Panel (g) shows the ratio of sequence tags mapping to the major (MaSat) relative to the minor (MiSat) satellites for wt and MCM2 deficient granulocyte (GRN), micronuclear (MN) and white blood cell (WBC) fractions as indicated.
(TIF)

S4 Fig Effect of MCM2 deficiency on 45S rRNA gene repeats. Panels a-f show MEFs from wt (a-c) and MCM2 deficient (d-f) embryos stained for nucleolin (a and d, red), γH2AX (b and e, green) and counter stained with DAPI (blue). Panels c and f are overlays of panels a/b and d/e respectively and the proportion of nucleoli containing γH2AX foci in wt (n = 122, 345) verses MCM2 deficient (n = 154, 148) cells are quantified for two experiments in panel g. Panels h and i compare sequence tag density over the 45S rRNA gene using data from total genomic DNA from thymus to estimate the ability to map sequences across the repeat (h) or short nascent strands prepared from wt (blue) or MCM2 deficient (red) MEFs by nascent strand capture and release (i) extracted from data in [12]. Panel (j) is a schematic representation of the repeat.
(TIF)

S5 Fig Comparison of wt, MCM2 deficient and HU treated MN sequence distributions. Panel a shows β and ρ values estimated from chr7 using pooled wt, MCM2 deficient and HU treated samples. To further estimate the accuracy of the estimation, bootstraps of 50% data points without replacement with 1000 repeats were carried out to estimate standard deviation and 95% confidence interval as shown in the table. Panel b shows normalized α values for individual wt (3), MCM2 deficient (2) and HU treated (2) samples. Panel c shows a plot of 45S ribosomal RNA gene (rDNA) sequence tag coverage (y-axis) against major satellite (MaSat) coverage for individual wt (3), MCM2 deficient (2) and HU treated (2) samples from both GRN fractions (G) and MN fractions (R). The shaded area indicates the 95% confidence interval excluding the MCM2 deficient MN samples.
(TIF)

S6 Fig Estimation of variance in γ values for Chr7 and Chr11. Chr7 and Chr11 were chosen to estimate experimental variance in γ values since by inspection they exhibit few local γ peaks. In panel a, γ value distributions are shown for each chromosome for hydroxyurea treated (HUEX), MCM2 deficient (Mcm2) and wild type (wt) mice as indicated. Panel b shows the standard deviations estimated from each of the distributions shown in panel a.
(TIF)

S1 Table Summary of sequencing. (TIF)

S2 Table HU γ-values at molecularly characterized mouse fragile site. (TIF)

S3 Table Proximal and distal 3 s.d. cutoff values for each chromosome under different conditions. (TIF)
