# Supplementary figures and images for: A Signature of Genomic Instability Resulting from Deficient Replication Licensing
Source: PLoS Genet. 2017 Jan 3;13(1):e1006547. doi: 10.1371/journal.pgen.1006547 (PMC5242545; doi:10.1371/journal.pgen.1006547)

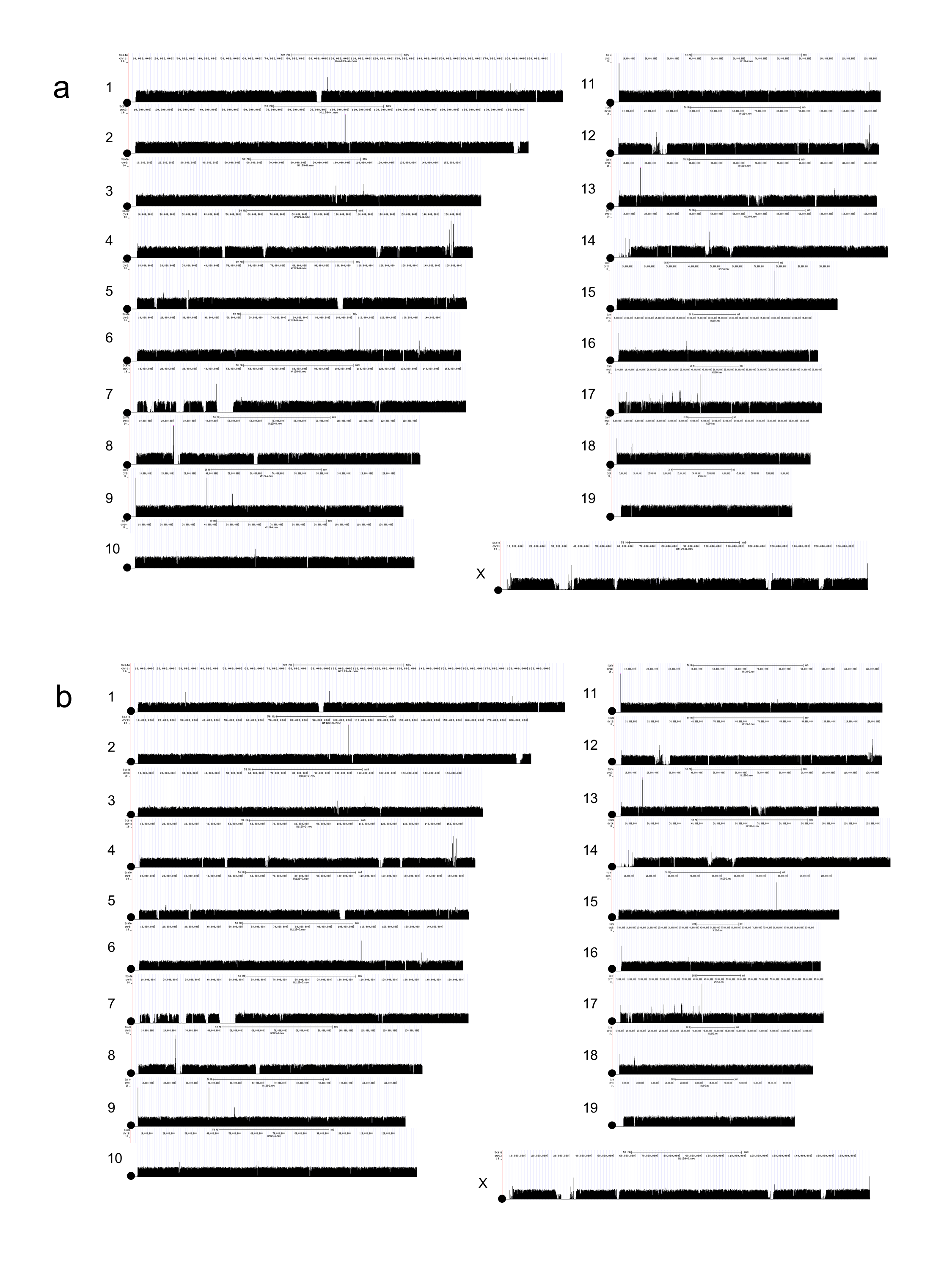

Supplement: S1 Fig — Tagged sequencing libraries were prepared from DNAs isolated from the WBC (panel a) and GRN (panel b) blood fractions of the 6 week old wt 129Sv mouse shown in Fig 1 for the MN fraction. Each whole chromosome is shown as indicated in the figures. The MN fraction shown in Fig 1a and the WBC and GRN fractions, shown in panels a and b here, were sequenced on the same lane using an Illumina HiSeq 2500 sequencer. (TIF) [file pgen.1006547.s001.tif]

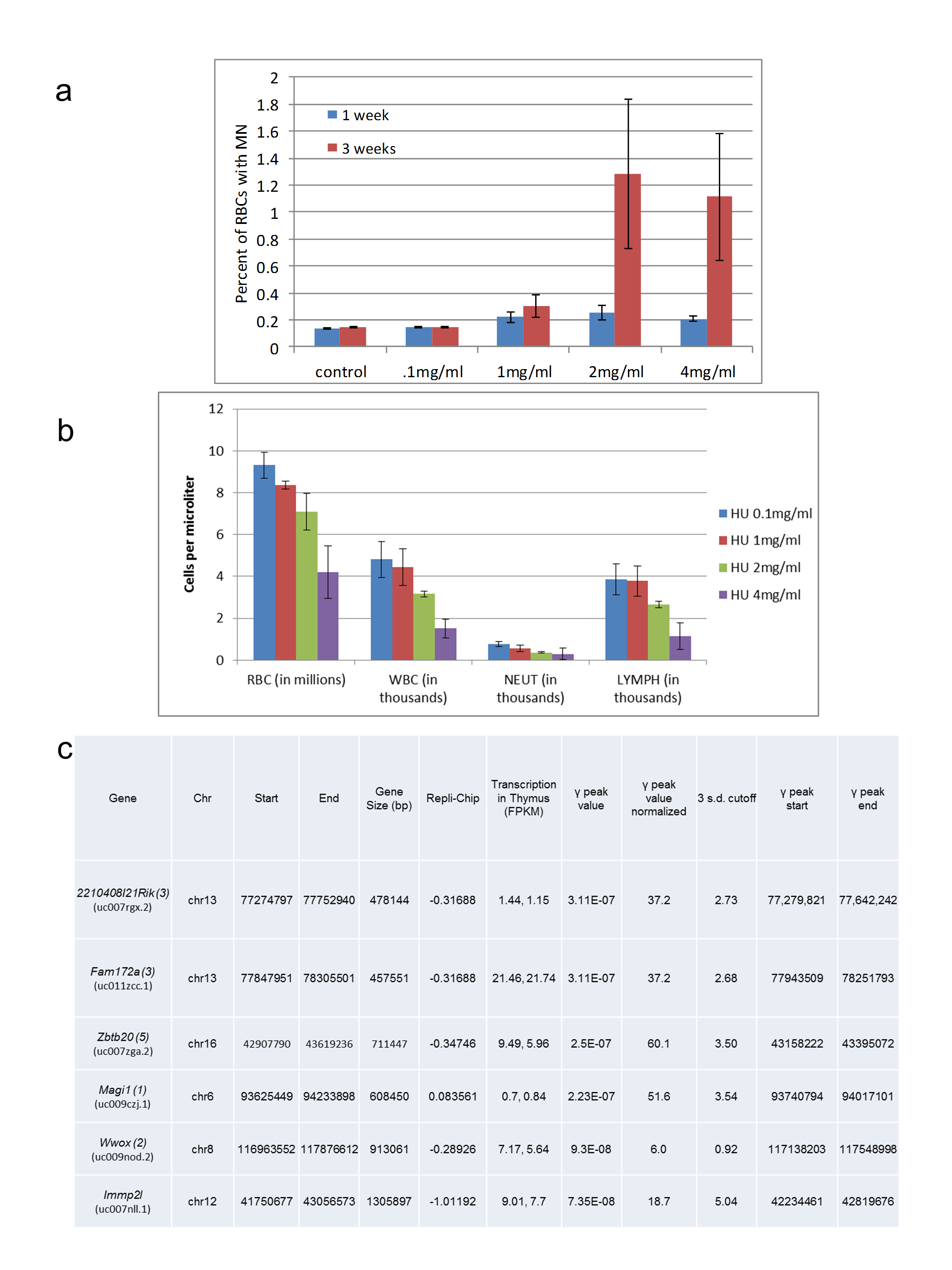

Supplement: S2 Fig — Wild type 129Sv mice were treated with varying concentrations of HU (n = 3 for each condition) as indicated and assayed for RBC MN frequency at 1 week or 3 weeks of treatment in panel (a) and by CBCs at three week of treatment in panel (b). Panel (c) is a list of genes over 300 kbp in length that contain the largest γ peak regions in HU treated mice. These include sites 1–3 and 5 (indicated in parentheses following the gene name) as marked in Fig 2 (site 4 lies within a segmental duplication on chr14 and is omitted since no gene is present). As shown in the table, the regions of instability identified by Mic-Seq lie within subdomains of the gene bodies. For each gene the gene coordinates, size in bp, replication timing value (FSU repli-ChIP for MEF; [31], FSU ENCODE group), relative transcription (fragments per kilobase of exon per million fragments mapped, FPKM, for two RNA seq replicates of thymus, [31], ENCODE LICR group), the local maximum for the γ value, the γ value normalized for β and ρ, the 3 s.d. cutoff value for the normalized γ value at the gene position, and the coordinates over which the change in slope occurs are given. (TIF) [file pgen.1006547.s002.tif]

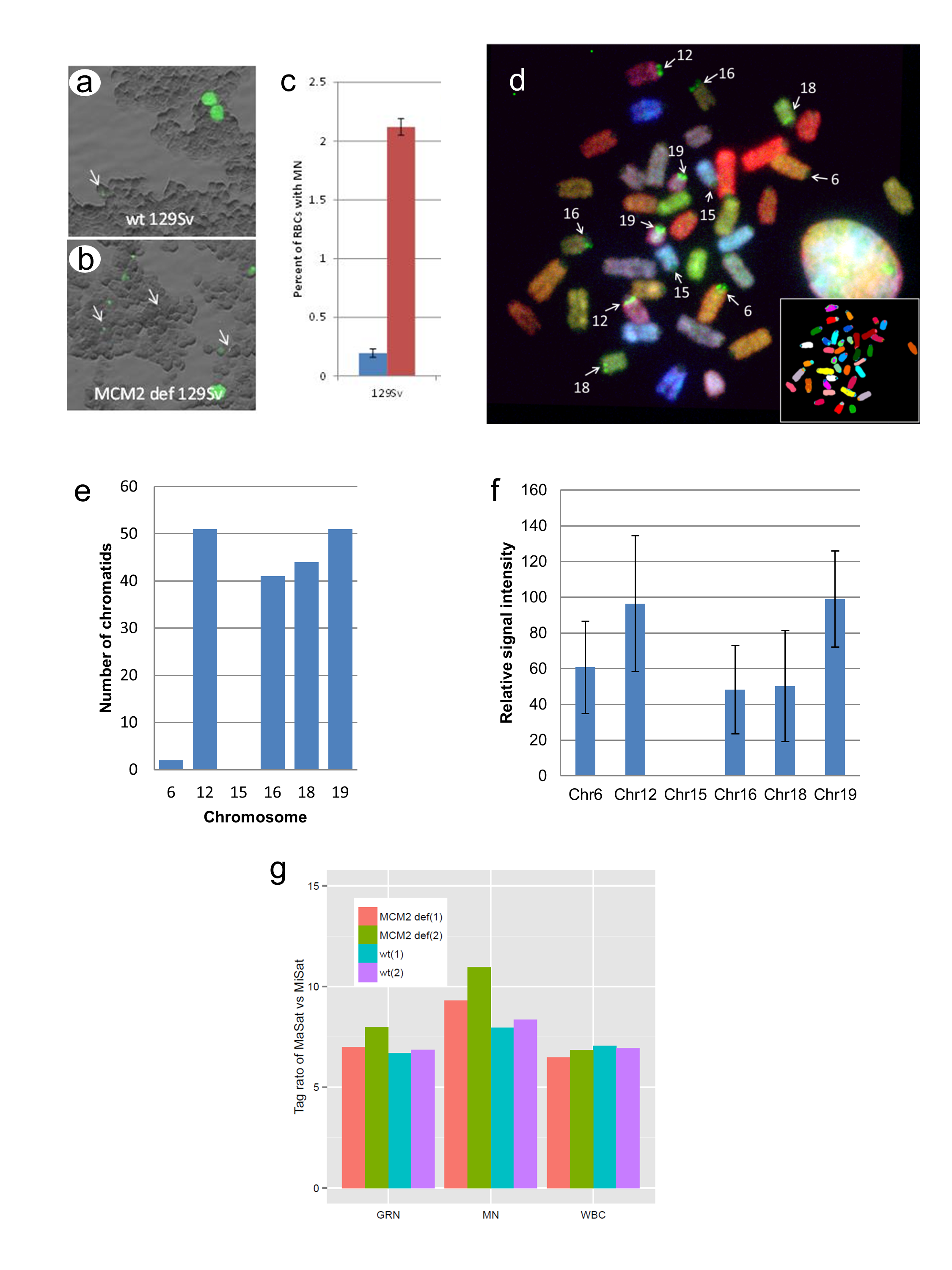

Supplement: S3 Fig — Panels A and B show 20X images of blood smears from 6 week old129Sv wt (panel a) and MCM2 deficient (panel b) mice stained with acridine orange (green). Arrows indicate micronuclei. Panel c shows RBC micronuclear frequency determined by flow cytometry for wt (blue, N = 2)) and MCM2 deficient (red, N = 2) 6 week old129Sv mice (error bars indicate s.d.). Panel d shows SKY/FISH analysis to identify chromosomes hybridizing to a 45S rRNA gene probe sequence where signal is seen as green (a spectral karyotype pseudo-colored of the larger image is shown in the inset). Chromosomes 6, 12, 15, 16, 18, and 19 are marked by arrows as indicated. Panel (e) shows the number of chromatids exhibiting 45S rRNA gene signal and panel (f) shows the average signal strength per chromatid (for those exhibiting signal) in 13 metaphase spreads (52 chromatids) for each chromosome (error bars indicate s.d.). Panel (g) shows the ratio of sequence tags mapping to the major (MaSat) relative to the minor (MiSat) satellites for wt and MCM2 deficient granulocyte (GRN), micronuclear (MN) and white blood cell (WBC) fractions as indicated. (TIF) [file pgen.1006547.s003.tif]

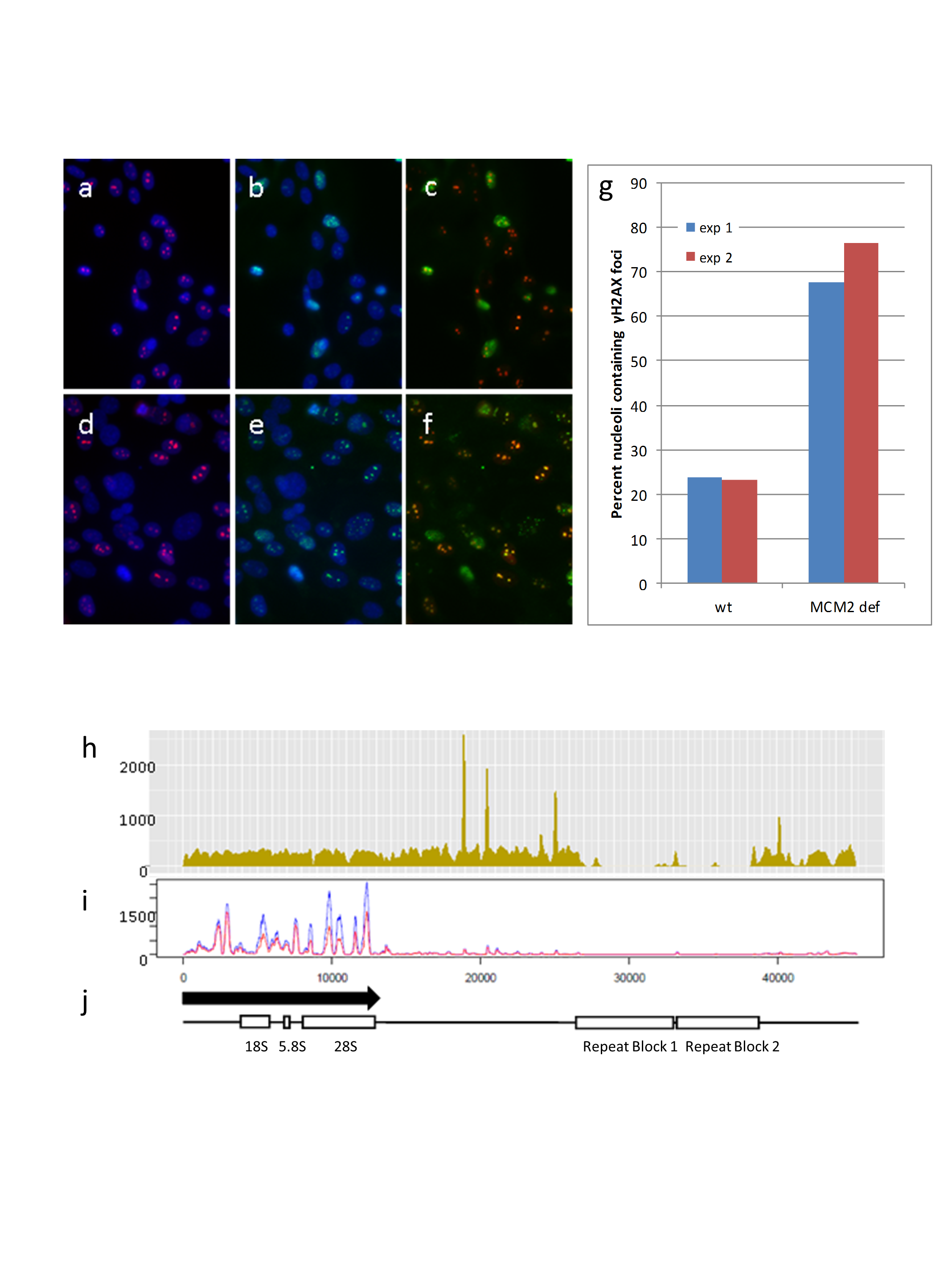

Supplement: S4 Fig — Panels a-f show MEFs from wt (a-c) and MCM2 deficient (d-f) embryos stained for nucleolin (a and d, red), γH2AX (b and e, green) and counter stained with DAPI (blue). Panels c and f are overlays of panels a/b and d/e respectively and the proportion of nucleoli containing γH2AX foci in wt (n = 122, 345) verses MCM2 deficient (n = 154, 148) cells are quantified for two experiments in panel g. Panels h and i compare sequence tag density over the 45S rRNA gene using data from total genomic DNA from thymus to estimate the ability to map sequences across the repeat (h) or short nascent strands prepared from wt (blue) or MCM2 deficient (red) MEFs by nascent strand capture and release (i) extracted from data in [12]. Panel (j) is a schematic representation of the repeat. (TIF) [file pgen.1006547.s004.tif]

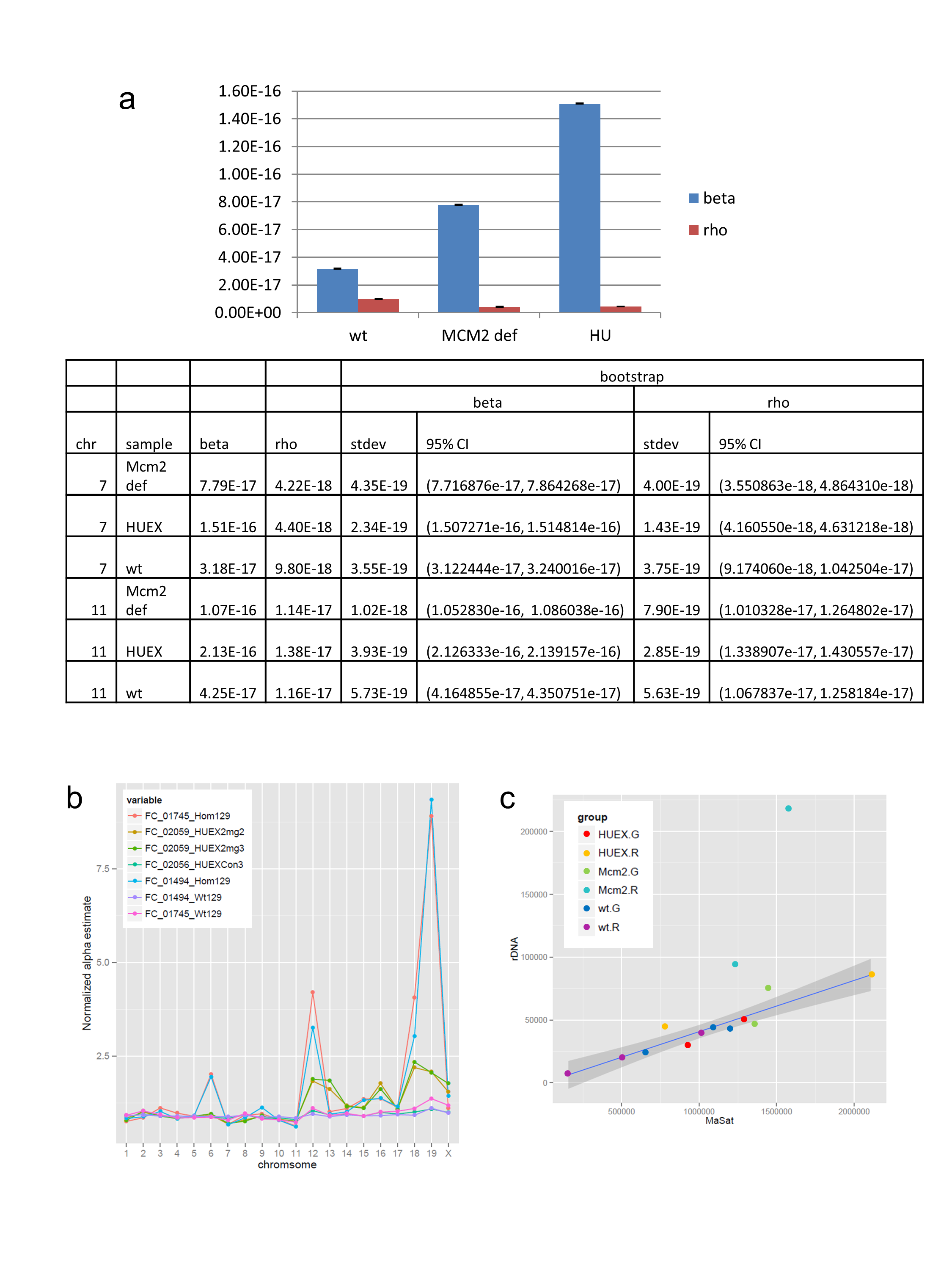

Supplement: S5 Fig — Panel a shows β and ρ values estimated from chr7 using pooled wt, MCM2 deficient and HU treated samples. To further estimate the accuracy of the estimation, bootstraps of 50% data points without replacement with 1000 repeats were carried out to estimate standard deviation and 95% confidence interval as shown in the table. Panel b shows normalized α values for individual wt (3), MCM2 deficient (2) and HU treated (2) samples. Panel c shows a plot of 45S ribosomal RNA gene (rDNA) sequence tag coverage (y-axis) against major satellite (MaSat) coverage for individual wt (3), MCM2 deficient (2) and HU treated (2) samples from both GRN fractions (G) and MN fractions (R). The shaded area indicates the 95% confidence interval excluding the MCM2 deficient MN samples. (TIF) [file pgen.1006547.s005.tif]

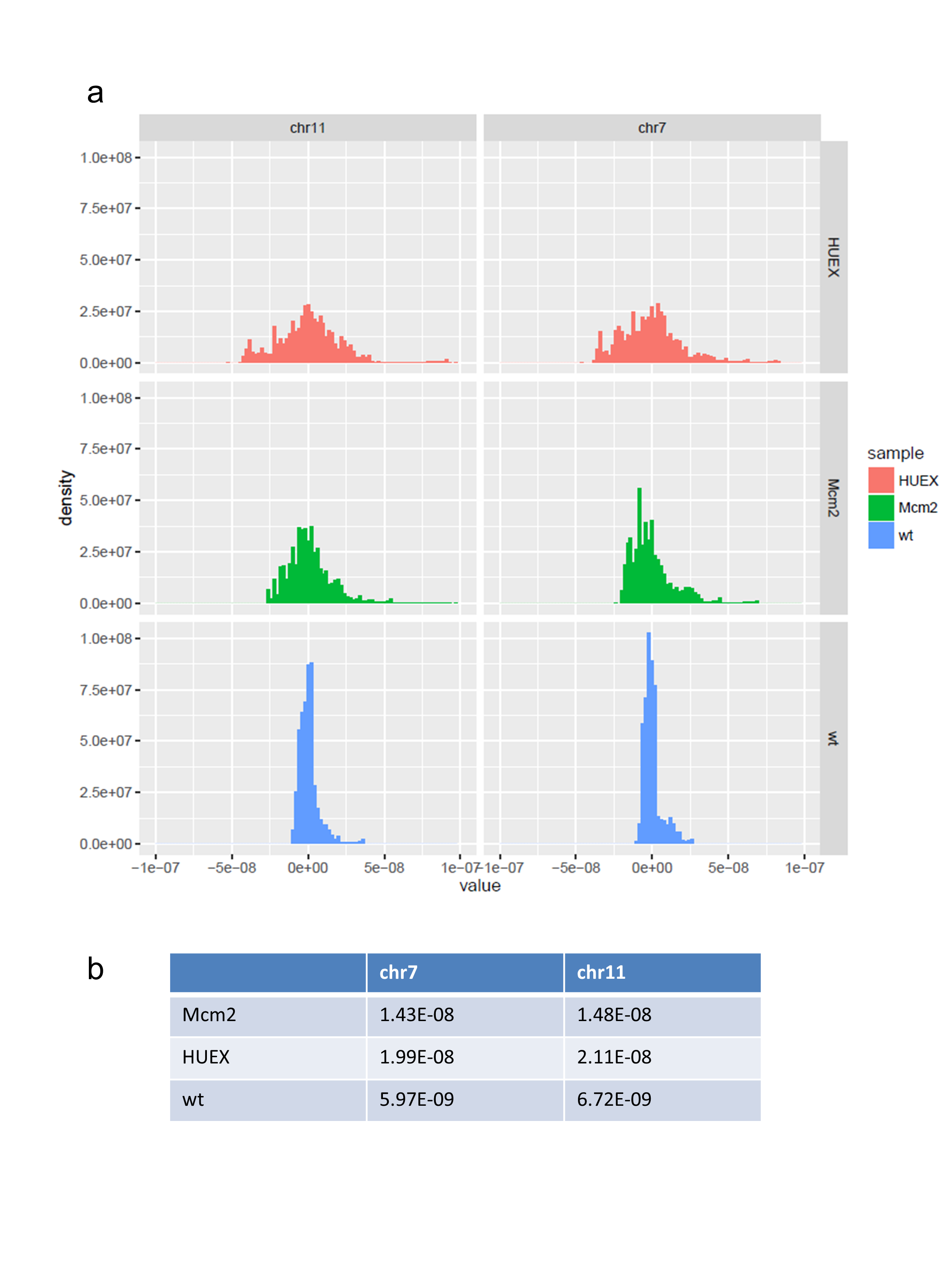

Supplement: S6 Fig — Chr7 and Chr11 were chosen to estimate experimental variance in γ values since by inspection they exhibit few local γ peaks. In panel a, γ value distributions are shown for each chromosome for hydroxyurea treated (HUEX), MCM2 deficient (Mcm2) and wild type (wt) mice as indicated. Panel b shows the standard deviations estimated from each of the distributions shown in panel a. (TIF) [file pgen.1006547.s006.tif]

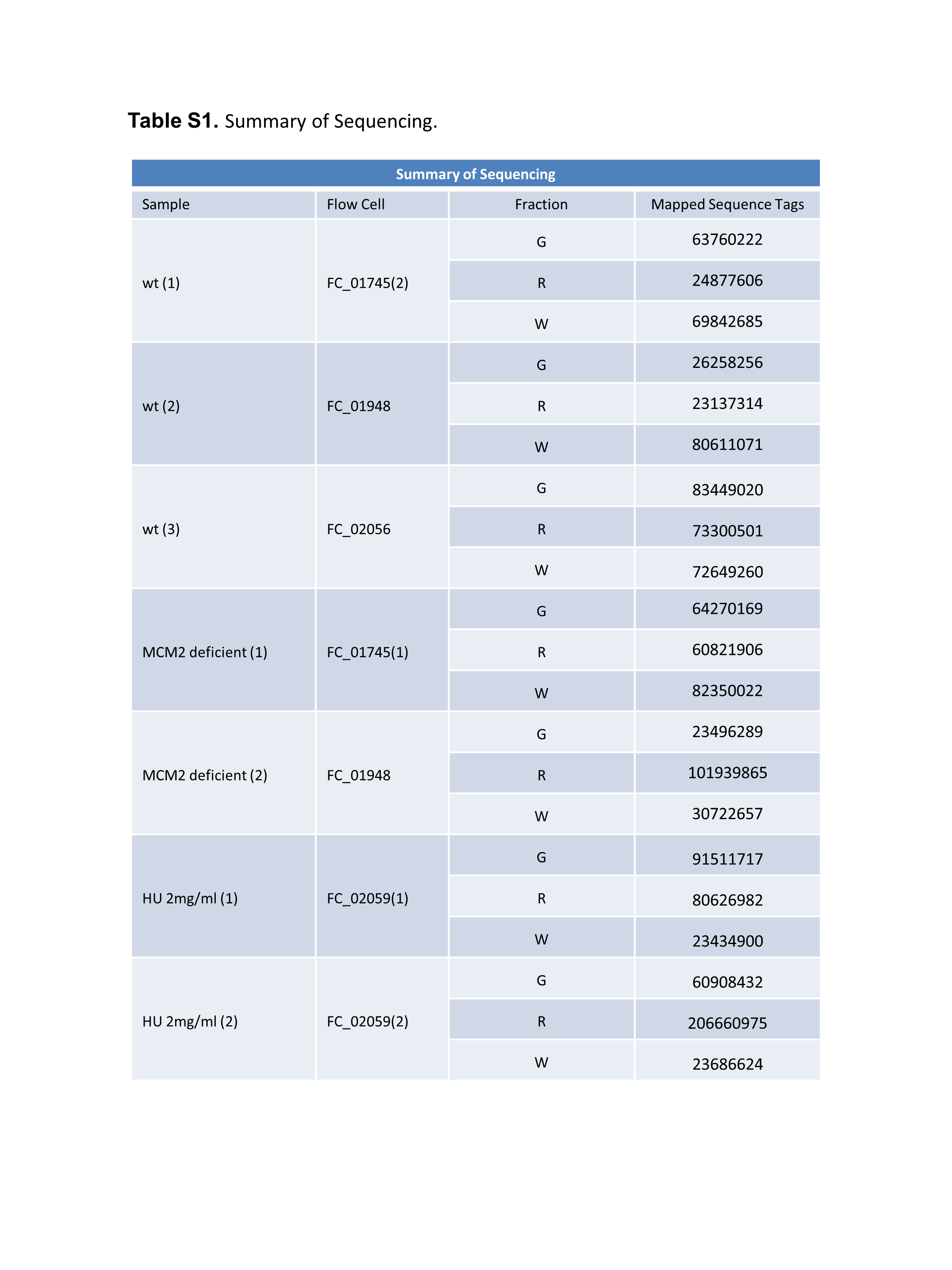

Supplement: S1 Table — (TIF) [file pgen.1006547.s007.tif]

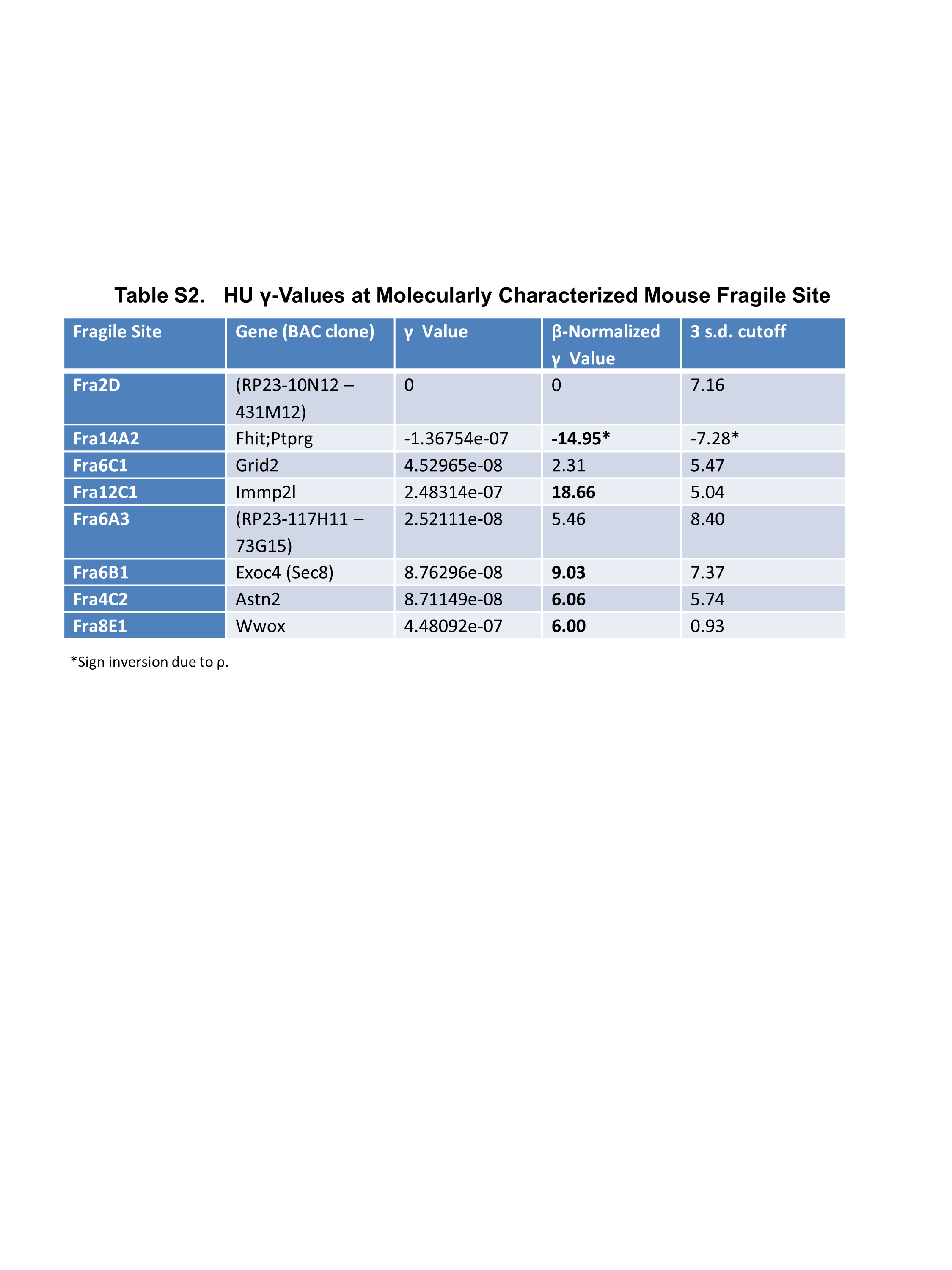

Supplement: S2 Table — (TIF) [file pgen.1006547.s008.tif]

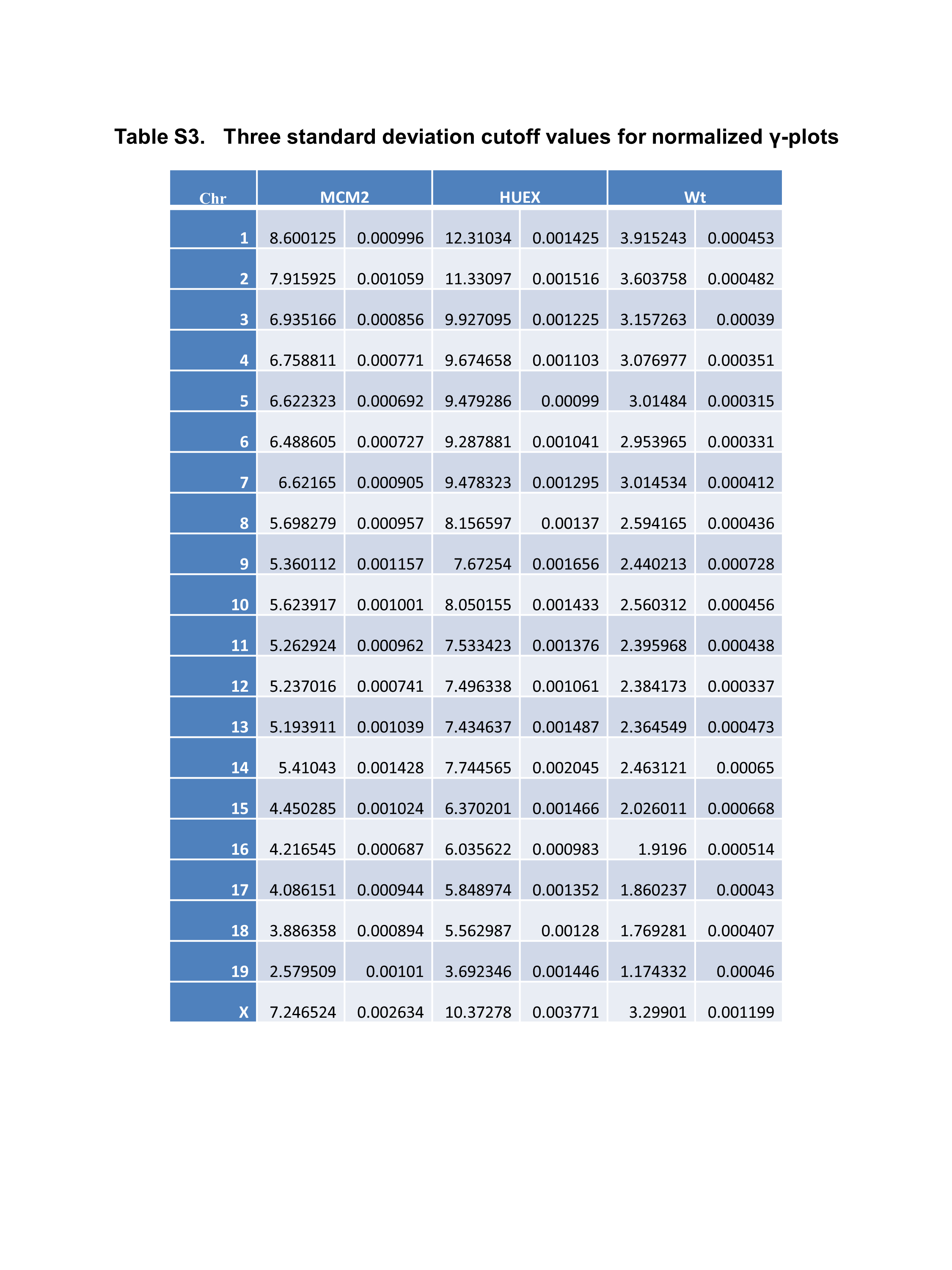

Supplement: S3 Table — (TIF) [file pgen.1006547.s009.tif]
